# Performance-contingent reward increases the use of congruent distracting information

**DOI:** 10.3758/s13414-023-02682-9

**Published:** 2023-03-14

**Authors:** Kerstin Fröber, Veronika Lerche

**Affiliations:** 1 Department of Psychology, University of Regensburg, Universitätsstr. 31, D-93053 Regensburg, Germany; 2 Department of Psychology, Kiel University, Kiel, Germany

**Keywords:** Simon, Proportion congruency effect, Congruency sequence effect, Drift diffusion model, Reward, Fast-dm

## Abstract

In conflict tasks like the Simon task, participants are instructed to respond to a task-relevant target dimension while ignoring additional distracting information. In the Simon task the distracting spatial information can be congruent or incongruent with the task-relevant target information, causing a congruency effect. As seen in the proportion congruency effect and the congruency sequence effect, this congruency effect is larger in mostly congruent blocks and following congruent trials, respectively. Common theories suggest that when the proportion of incongruent trials is high or after an incongruent trial, focus on the task-relevant target information is increased and distracting information is inhibited. In two experiments, we investigated how reward modulates these phenomena. Specifically, performance-contingent reward – but not non-contingent reward – increased the usage of the distracting information in mostly congruent blocks or following congruent trials, while the adaptation to incongruency (i.e., mostly incongruent blocks or preceding incongruent trials) was the same in all conditions. Additional diffusion model analyses found that this effect of performance-contingent reward was captured by the drift rate parameter. These results suggest an increased focus on the target information by incongruent trials independent from reward, while the adaptation to (mostly) congruent trials characterized by increased usage of distracting information can be motivationally boosted. That is, performance-contingent reward increases the use of congruent distracting information beyond a mere relaxation of the increased target-focus following (mostly) congruent trials.

## Introduction

Rewards – contingent or non-contingent on performance – are omnipresent in our every-day life. For example, in the Olympic Games, the best performing athlete is rewarded with the gold medal (performance-contingent reward), whereas the first drawing of a child is rewarded by the parents irrespectively of the artistic quality (non-contingent reward). Increasing evidence suggests that these two kinds of reward have differential influence on cognitive control processes (for reviews, see Chiew, 2021; Dreisbach & Fischer, 2012; Dreisbach & Fröber, 2019; Notebaert & Braem, 2016).

In the present study we aim to gather more evidence for this by investigating how performance (non-)contingent reward influences context processing in a conflict task. As reviewed below, reward effects on context processing have been well studied using the AX-continuous performance task (AX-CPT), but the literature on the influence of performance (non-)contingent reward prospect on context processing in conflict tasks is characterized by mixed results so far. Therefore, the present study is aimed to shed more light on motivational modulations of context processing in conflict tasks by focusing on two phenomena of adaptive control – the proportion congruency effect and the congruency sequence effect. To this end, we present two high-powered experiments and extend the behavioral analyses by drift diffusion modeling. By focusing on control adaptations in conflict tasks and analyzing data with the drift diffusion model, our study can furthermore provide new insights on the dynamic mechanisms of target enhancement and distractor suppression in conflict tasks.

### How performance (non-)contingent reward modulates context processing in the AX-continuous performance task

As seen in the initial every-day example, reward can be provided contingent or non-contingent on performance. Performance-contingent reward means that a certain performance criterion has to be met to get a reward. Non-contingent reward, on the other hand, is given unconditionally as a gift and irrespectively of current performance. Furthermore, anticipatory effects of reward (reward prospect) often differ from aftereffects of reward (reward as feedback). That is, the announcement and expectation of an upcoming reward can have differential effects from the recent receipt of a reward. Thus, Notebaert and Braem (2016) aimed to parse the effects of reward on cognitive control by differentiating between an affective, a motivational, and a learning component of reward. Each of these components was associated with a specific effect on cognitive control. While performance-contingent reward *as feedback* is assumed as a signal that increases reinforcement learning and promotes exploitation, performance-contingent reward *prospect* is supposed to have a motivational effect that increases preparatory, sustained control. Finally, non-contingent reward (as feedback and prospect) primarily induces positive affect, which is associated with a relaxation of attentional focus and increased flexibility.

In line with this differentiation, it has been found that performance-contingent reward prospect increases the reliance on informative (i.e., useful, but not 100% valid) context cues for response preparation (Chiew & Braver, 2013, 2014; Locke & Braver, 2008), whereas non-contingent reward prospect reduces the usage of such context cues (Fröber & Dreisbach, 2014, 2016a). This was found using the AX-CPT, a context-processing task in which participants have to respond to a probe letter depending on the context set by a preceding cue letter. A target response is required whenever a probe letter X follows the cue letter A (70% of all trials), whereas a different, non-target response is required in all other cases (AY trials: a letter other than X follows cue A; BX trials: an X follows another cue letter than A; BY: neither A nor X are presented as cue and probe letter; all three trial types are presented with a probability of 10% each). In the performance-contingent reward condition, participants had to be accurate and especially fast to get a reward (Fröber & Dreisbach, 2014, 2016a). This led to a motivational effect in terms of increased use of the cue letter for response preparation. Consequently, performance benefits were found in AX, BX, and BY trials, but error rates selectively increased in AY trials, where the expectation of an X following the A cue was violated. When participants were provided with non-contingent reward irrespective of performance, the context information of the cue letter was less used for response preparation with oppositional behavioral consequences, that is, improved performance specifically in AY trials. Using different versions of this task, Hefer and Dreisbach could show that the increase in preparatory control under performance-contingent reward prospect generalizes to any predictive context information and is accompanied by reduced flexibility in terms of delayed adaptation to changed task or reward conditions (Hefer & Dreisbach, 2016, 2017, 2020a). That is, performance-contingent reward prospect seemed to boost a specific mode of cognitive control in the AX-CPT with increased maintenance and reliance on informative context cues to optimize response preparation (in the literature often called *proactive control*, cf., Braver, 2012).

In the AX-CPT, this cognitive control mode is very efficient, because the context information provided by the cue letter is highly predictive (90%) for the required response to the subsequent target letter. Therefore, even without external rewards, participants show a shift towards increased cue usage with increasing time on task (Fröber & Dreisbach, 2016a; Hefer & Dreisbach, 2020b). This suggests that this strategy is in itself quite rewarding in the AX-CPT, but it can be further boosted by performance-contingent reward prospect. However, context information is not in all situations that useful, and increased and sustained reliance on context information can then be detrimental to performance. Even in the AX-CPT, increased usage of the context cue letters results in selectively increased performance costs in the rare cases (AY trials; 10% of all trials), where the cue information is misleading for the required response. Here, the increased reliance on the context information creates a conflict in terms of a cue validity effect, resulting in increased error rates and reaction times (RTs). In many everyday situations and other experimental paradigms contextual information is less informative than in the AX-CPT, thereby frequently challenging the pursuit of an intended task goal by conflict between task-relevant target information and task-irrelevant – but pre-dominant – distractor information. Consequently, sustained increased usage of context information is not the best strategy and instead dynamic adaptations of cognitive control are needed with more or less reliance on the context information.

### Control adaptations in conflict tasks as adaptations in context processing

In the lab, situations with task-relevant target information and task-irrelevant distractor information are investigated with selective attention tasks like the Stroop task (Stroop, 1935), the Eriksen flanker task (Eriksen & Eriksen, 1974), or the Simon task (Simon & Rudell, 1967). Trials where the target information and the distractor information are associated with different responses are called incongruent, and trials with corresponding target and distractor information are called congruent. Processing of the distracting context information is beneficial in congruent trials, but it conversely causes interference in incongruent trials; the difference between the two is called congruency effect.

In these conflict tasks, context processing is usually less compelling than in the AX-CPT, where context processing is inherently promoted by the cue letters in the beginning of a trial. Nonetheless, two well-replicated phenomena of adaptive control,^1^ namely the proportion congruency effect (PCE) and the congruency sequence effect (CSE), suggest that context processing plays an important role in these tasks, too (for a recent review, see Braem et al., 2019). To be more precise, the PCE refers to the finding that the congruency effect is smaller in blocks of mostly incongruent trials compared to blocks of mostly congruent trials (for a review, see Bugg & Crump, 2012). And the CSE – typically assessed in a setting with equal probability of congruent and incongruent trials – describes control adaptations from trial to trial with a smaller congruency effect following incongruent trials versus congruent trials (for reviews, see Duthoo et al., 2014; Egner, 2007). That is, one can observe that depending on the context in terms of the proportion of congruency of a whole list of trials or the congruency of an immediately preceding trial, more or less of the distracting information seems to be processed together with the target information.

As early as three decades ago, Gratton et al. (1992) systematically investigated the CSE and PCE in a Flanker task, and suggested that participants optimize the use of context information by adaptively changing the weight given to different modes of processing. They assumed that responses in this task can be based either on evidence accumulated during an early phase of parallel processing, where both task-relevant target information and distractor information are processed together, or a later phase of focused processing with enhanced focus on the target and inhibition of the distractors. Gratton et al. suggested that participants strategically make more use of context processing (increased weight on the parallel phase) after congruent trials and in blocks of mostly congruent trials, and rely more on focused processing after incongruent trials or in blocks of mostly incongruent trials. That is, the weight given to the different processing modes is adapted according to expectancies based on the congruency of the previous trial(s). This optimizes performance in terms of maximizing the facilitation in congruent trials and minimizing the interference in incongruent trials. While such an account seems especially plausible for PCE effects where expectancies based on the high frequency of (in)congruent trials seem likely, Gratton et al. assume that participants expect repetitions of the previous trial type even if the objective probability for another (in)congruent trial is 50%. That is, the same explanation is also used to explain the CSE.

About 10 years later, Botvinick et al. (2001) developed the influential conflict monitoring theory (CMT) to explain control adaptations like the CSE and the PCE. They extended the empirical work of Gratton et al. with computational model simulations based on neuroscientific evidence. In the CMT, the anterior cingulate cortex serves as a conflict monitoring unit that detects conflicts in information processing in terms of simultaneous activation of incompatible representations (incongruent target and distractor information). The detection of such a conflict in an incongruent trial then elicits a control signal that increases the focus on the target via a feedback-loop and thereby diminishes the impact of the distractor in information processing. Simulations using this model could successfully generate the typical empirical data patterns: Interference by the distractor in an incongruent trial, but also facilitation by the distractor in a congruent trial was reduced following an incongruent trial (CSE). Furthermore, the PCE could be successfully simulated by a cumulative effect of more frequent incongruent trials. Confirming the control adaptation mechanism assumed in the CMT, Egner and Hirsch (2005) provided further neuroscientific evidence in terms of cortical amplification of target information – and not inhibition of distractor information – following incongruent trials in a Stroop task. Note, however, that studies using the Simon task found evidence in lateralized readiness potentials for distractor suppression underlying the CSE (Stürmer et al., 2002; Stürmer & Leuthold, 2003).

Another 10 years later, Schlaghecken and Martini (2012) challenged the conflict monitoring account with its focus on conflicts as triggers of control adaptations, and suggested a general context adaptation mechanism as an alternative explanation for the CSE and PCE. Similar to the expectation account of Gratton et al. (1992), they suggested that the responsiveness to distractor information (i.e., the activation of the response channel) and the output threshold in the visuomotor system (i.e., the threshold of activation that needs to be reached for response execution) is adjusted with respect to the preceding congruency with a functional role of both incongruent and congruent trials. To be more precise, the system is assumed to relax following congruent trials, resulting in increased early, distractor-related activation and a lowered output threshold. Conversely, the shielding against distraction is increased following incongruent trials, causing decreased distractor-related activation and a higher output threshold. In line with such a general context adaptation mechanism, Jiménez and Méndez (2013; Jiménez & Méndez, 2014) demonstrated a cumulative, sequential adaptation effect to previous (in)congruency, while controlling for the influence of low-level feature priming effects. Interestingly, these authors could demonstrate a dissociation of this effect from explicit expectations, suggesting a more automatic source of this adaptation.

Taken together, context processing plays an important role in conflict tasks and is subject to dynamic adaptations, but the exact mechanism underlying these adaptations is still a matter of debate. Besides the just reviewed explanations, many more accounts with or without the assumption of cognitive control processes exist (for reviews, see Braem et al., 2019; Egner, 2007, 2014). In the present study, we aim to investigate how dynamic adaptations of context processing in conflict tasks interact with (non-)contingent reward prospect, thereby potentially gaining more insight into the underlying mechanisms. For example, if there is indeed a general context adaptation mechanism as suggested by Schlaghecken and Martini (2012), this adaptation should be further boosted by performance-contingent reward prospect and relaxed by non-contingent reward prospect, similar to previous results from AX-CPT studies that demonstrated a modulation of the use of context information by (non-)contingent reward (Fröber & Dreisbach, 2014, 2016a).

### How performance (non-)contingent reward modulates control adaptations

First studies investigating the influence of (non-)contingent reward on control adaptations in conflict tasks focused on reward as feedback only. Using the Flanker and the Simon tasks, these studies found differential effects of performance-contingent and non-contingent reward on the CSE (for a review, see Dreisbach & Fischer, 2012). When reward receipt was dependent on fast and correct responding, the CSE was increased following rewarded trials. Depending on the study, this moderation by reward was found for both trials following congruent and trials following incongruent trials (Stürmer et al., 2011) or specifically for trials following incongruent trials (Braem et al., 2012). In contrast, when rewards were given randomly and irrespective of performance, the CSE was reduced, with an impact specifically on trials following incongruent trials (van Steenbergen et al., 2009, 2012), or no influence on the CSE was found (Stürmer et al., 2011). All in all, these results seem to fit with the dissociation between performance-contingent and non-contingent reward seen in the AX-CPT studies reviewed above. However, a direct comparison is not unproblematic because the AX-CPT studies used reward prospect manipulations whereas the conflict studies used reward as feedback manipulations. And indeed, the literature so far on (non-)contingent reward *prospect* and control adaptations is heterogeneous and rather inconclusive. Most studies on reward effects in conflict tasks focused on performance-contingent reward only and some did not investigate control adaptations, but only targeted the congruency effect per se (e.g., Padmala & Pessoa, 2011; van den Berg et al., 2014).

Two studies targeting the interplay of performance-contingent reward prospect and control adaptations were done by Soutschek et al. (2014, 2015). Using a Stroop-like task, Soutschek et al. (2014) found that performance-contingent reward prospect influenced the PCE (Experiment 2), but not the CSE (Experiment 1). In Experiment 2, the authors found that without reward prospect the congruency effect was smaller in mostly incongruent blocks (typical PCE), but with reward prospect the congruency effect was equally small in both mostly congruent and mostly incongruent blocks. The authors concluded that both reward prospect and a high proportion of incongruent trials increased the focus on the target information without an additional improvement if both come together. Note that reward prospect in this study was intermixed with loss prospect, since participants could gain a reward after blocks with high accuracy and fast mean RTs, while blocks with low accuracy or slow mean RTs were punished by a loss. Using the same reward manipulation in a follow-up study including functional magnetic resonance imaging (fMRI), Soutschek et al. (2015) found neuroscientific evidence that reward prospect improved conflict processing by amplifying the target information, whereas the PCE was associated with a modulation of distractor information.

What could be problematic in the studies by Soutschek et al. (2014, 2015) is the combined manipulation of reward and loss prospect. Using a Simon task, Choi and Cho (2020) directly compared the influence of reward prospect (Experiment 1) and loss prospect (Experiment 2) – manipulated in a blocked manner – on the congruency effect and the CSE. In Experiment 1, prospect of a large reward compared to prospect of a small reward did not influence the congruency effect per se but reduced the CSE: After congruent trials the congruency effect was smaller in the large reward compared to the small reward condition and after incongruent trials the congruency effect was larger in the large reward compared to the small reward condition. In contrast, in Experiment 2, prospect of a large versus small *loss* increased the congruency effect per se, but did not affect the CSE. These results suggest a dissociation between approach (reward) and avoidance (loss) motivation. Dissociations in modulations of the CSE by gains and losses have also been found in the reward as feedback literature (Stürmer et al., 2011; van Steenbergen et al., 2009, 2012).

To the best of our knowledge, only one study so far targeted the differentiation between performance-contingent and non-contingent reward prospect, and directly compared the effects of reward prospect and aftereffects of reward receipt, although this differentiation is theoretically of high importance (cf., Notebaert & Braem, 2016). Yamaguchi and Nishimura (2019) presented reward cues on single trials of a Flanker task without allowing direct repetitions of reward trials. In a first experiment, accurate responses in these trials were followed by a gain, whereas errors were punished by a loss. So, like in the Soutschek et al. (2014, 2015) studies, reward prospect was intermixed with loss prospect. In two further experiments reward cues announced a random gain or random loss irrespective of performance, with a higher probability of gains (Experiment 2) or losses (Experiment 3). In Experiment 1, results showed a reduction of the congruency effect per se by performance-contingent reward prospect compared to trials without reward prospect, but no effect of reward feedback on the CSE. Non-contingent reward prospect (Experiment 2) did not modulate the congruency effect per se, but the CSE was reduced after reward feedback due to less adaptation after incongruent trials. Finally, non-contingent loss prospect (Experiment 3) had no significant influence on the congruency effect or the CSE, but reduced general RTs in the current trial and slowed down RTs in the following trial.

Taken together, the literature so far is quite mixed in results and characterized by differences in the targeted control adaptation phenomena and inconsistencies with respect to the reward (prospect) manipulations. While there is some evidence for dissociations between performance-contingent and non-contingent reward on control adaptations in conflict tasks, it is hard to draw strong conclusions about modulations of context processing by reward prospect in conflict tasks based on the existing evidence. Therefore, in the present study, we will use a pure manipulation of reward prospect – that is, without mixing reward and loss prospect – and we will directly compare performance-contingent and non-contingent reward prospect within participants.

### The present study

The aim of the present study was to investigate the effect of performance (non-)contingent reward prospect on context processing in a conflict task. Systematic research on this question has already been done with the AX-CPT paradigm (Fröber & Dreisbach, 2014, 2016a), whereas the literature on effects of reward prospect on context processing in conflict tasks is very heterogenous and characterized by mixed results so far. In conflict tasks, context information – that is, task-irrelevant distractor information – is typically lower in demand characteristics than the cue letters in an AX-CPT. Furthermore, the usage of distracting context information is subject to dynamic adaptions like the PCE and the CSE. Based on the converging evidence from the AX-CPT literature (Chiew & Braver, 2013, 2014; Fröber & Dreisbach, 2014, 2016a; Hefer & Dreisbach, 2016, 2017, 2020a; Locke & Braver, 2008), we expect that performance-contingent reward prospect increases the usage of context information for response preparation, whereas non-contingent reward should be associated with less reliance on context information.

To this end, we focused on two control adaptation phenomena, namely the PCE and the CSE. To elicit a PCE, a high frequency of (in)congruent trials was used in a given block of trials. As a consequence, context information by the task-irrelevant distractor should be similarly predictive to the cue letters in an AX-CPT. If performance-contingent reward indeed motivates to increase the use of this predictive context information, participants should show increased facilitation on congruent trials in a mostly congruent block. This should be accompanied by increased interference in the rare cases of an incongruent trial similar to the selectively increased error rate in misleading AY trials in the AX-CPT. Increased usage of the context information is not helpful in mostly incongruent blocks, so that target processing is typically enhanced and distractor information suppressed. Results will show whether this is further boosted by performance-contingent reward prospect and/or relaxed by non-contingent reward prospect.

In conflict tasks with an equal proportion of congruent and incongruent trials, participants typically show a similar control adaptation – the CSE – in terms of reduced interference but also reduced facilitation effects after incongruent trials. In this case, congruency in the previous trial is not predictive of the upcoming congruency, but some accounts suggest a general context adaptation mechanism underlying both the PCE and the CSE. Thus, performance-contingent reward prospect might likewise increase facilitation and interference effects after congruent trials due to increased usage of distracting context information even though the probability of another congruent trial is unpredictable. Conversely, non-contingent reward prospect might relax the control adaptation typically found after incongruent trials.

Taken together, we expect to find an increased PCE and probably CSE under performance-contingent reward prospect, but a reduced PCE and CSE under non-contingent reward as compared to an additional neutral condition without any reward prospect. As a conflict task, we used a number Simon task with digits from 1 to 9 (excluding 5) presented left or right from the screen center. Participants were instructed to respond to digits smaller than 5 with a left-hand response and to digits larger than 5 with a right-hand response. Conflict in this task emerges by the overlap between target and response location, and is further boosted by a SNARC effect (spatial numerical associations of response codes; Dehaene et al., 1993). That is, this task combines two types of stimulus-response conflict.

We conducted two experiments. In both of them, participants started with a first phase without reward manipulation to check whether the control adaptation phenomena of interest (Experiment 1: PCE; Experiment 2: CSE) were present in this task. In a second phase, we introduced three reward prospect conditions that varied in a blocked manner. In the performance-contingent reward prospect condition, participants were provided with rewards for responses which were both accurate and especially fast without punishing errors or slow responses with a loss. In the non-contingent reward condition, reward was given in every trial as a gift, irrespectively of the current performance and thus even for errors. In addition, we had a neutral control condition without prospect of reward.

By definition, performance-contingent reward requires that participants meet a certain performance criterion to get a reward. This performance criterion has to be challenging for the individual participants, because otherwise the reward can be perceived as an easy gain and elicit non-contingent reward effects instead (cf., Müller et al., 2007). To implement this in the lab, rewards are typically provided for responses that are both accurate and especially fast with individually determined response thresholds. A common critique on this procedure is that it causes a speed-accuracy trade-off instead of a true performance optimization. While several studies provide evidence for performance-contingent reward effects beyond mere shifts in response criterion,^2^ we decided to directly address this issue by analyzing our data not only in terms of mean RTs and error rates, but additionally with the drift diffusion model (DDM).

**Fig. 1:**
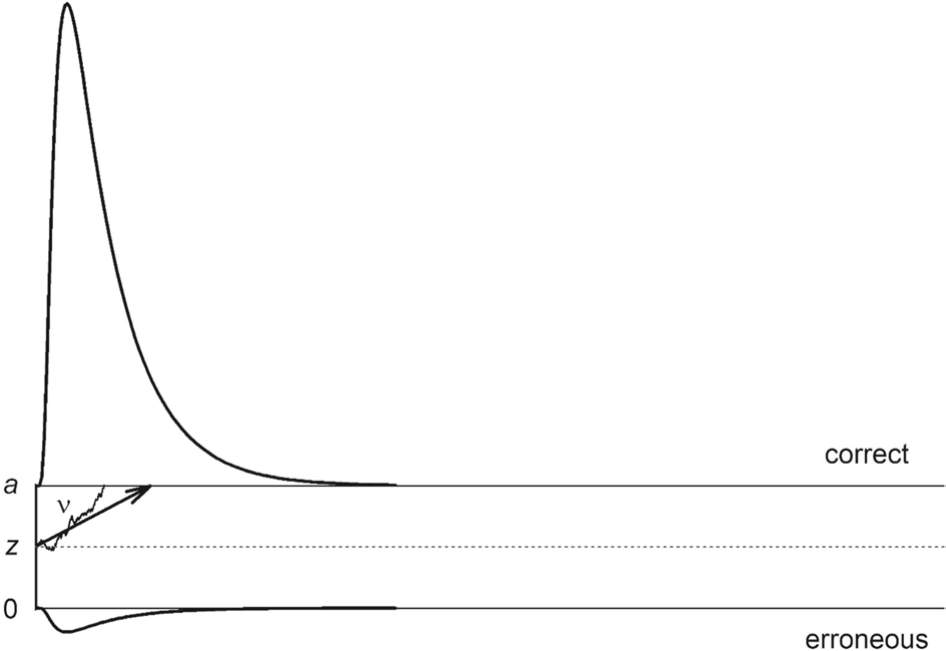
Exemplary diffusion model trial. *Note.* In this example, the thresholds are associated with correct and erroneous responses and the starting point is positioned at the center between the thresholds. The exemplary trial ends at the correct threshold. The upper threshold is approached with drift rate *ν*. Non-decision time is not depicted in the figure

The DDM (Ratcliff, 1978) belongs to the class of evidence accumulation models (for reviews, see Evans & Wagenmakers, 2020; Ratcliff et al., 2016; Voss, Nagler, & Lerche, 2013a) and is typically applied to very fast binary decision tasks (but see, e.g., Lerche & Voss, 2019 for an exemplary application to an RT task that takes several seconds per trial). The DDM assumes that information is accumulated continuously until at some point one of two thresholds has been reached. Figure 1 shows an exemplary trial. In the figure, the thresholds are associated with correct and erroneous responses (i.e., so-called accuracy coding). The accumulation process starts in the middle between the two thresholds; thus, the starting point (parameter *z*) is centered. With a centered starting point, there is no prior bias for either of the two response options of the task before the stimulus is shown. To the continuous information uptake that is mapped by the parameter drift rate (ν) random noise is added (i.e., the diffusion coefficient that is fixed in parameter estimation), resulting in a wiggly path, which in the exemplary trial ends at the upper threshold.

The drift rate is a measure of speed of information accumulation with higher values representing higher speed of information accumulation. Higher drift rates go along with lower error rates and lower mean RTs, with a larger association of drift rate with error rate than with mean RT (Ratcliff & McKoon, 2008). Drift rates depend on characteristics both of the task (larger values for easier tasks; e.g., Ratcliff, 2008; Voss et al., 2004) and of the participant (larger values for more intelligent participants; e.g., Lerche et al., 2020; Schmiedek et al., 2007). As noted, one main reason for applying the DDM in our experiments was the fact that it provides a measure of a speed-accuracy trade-off. Speed-accuracy trade-offs are represented by the distance between the two thresholds, the so-called threshold separation (*a*). If the focus is on accuracy, the threshold separation is larger, indicating that participants accumulate more information before making a decision. This, in turn, results in prolonged RTs, but at the same time fewer errors. Thus, higher values of threshold separation go along with lower error rates and higher mean RTs. The association of threshold separation with error rate is smaller than with mean RT (Ratcliff & McKoon, 2008).

If, on the other hand, the focus is on speed, threshold separation is smaller, responses are less accurate, but also faster. Like drift rate, threshold separation also depends on characteristics both of the task (e.g., higher threshold separation values in conditions with accuracy instructions, Ratcliff, 2008; Voss et al., 2004) and of the participant (e.g., larger threshold separation values for older participants; e.g., von Krause et al., 2022; Theisen et al., 2020). In addition to the actual decision process, participants also have to encode the information and execute the motoric response. Such processes are mapped by the non-decision time component of the diffusion model (*t*_*0*_). Non-decision time also depends on task characteristics (e.g., it has been found to be larger in tasks requiring a more complex motoric response; Lerche & Voss, 2019; Voss et al., 2004) and on the participant (e.g., larger non-decision time values for older participants; von Krause et al., 2022; Theisen et al., 2020). Several other parameters have been included in extended versions of the diffusion model (e.g., intertrial variabilities; Ratcliff & Rouder, 1998; Ratcliff & Tuerlinckx, 2002). Notably, the main parameters of the diffusion model (ν, *a*, *t*_*0*_, *z*) have also been validated by means of experimental validation studies (e.g., Arnold et al., 2015; Lerche & Voss, 2019; Voss et al., 2004).

We decided to use the DDM because it offers the opportunity to directly investigate a potential speed-accuracy trade-off that might be caused by a manipulation of reward prospect and because the model disentangles such a trade-off from other processes involved in task execution. Accordingly, the DDM parameters can be seen as purer measures of cognitive processes than the behavioral variables mean RT and error rate. The standard diffusion model or extensions thereof have already been successfully applied to conflict tasks (e.g., Aschenbrenner, 2016; Elliot & Aarts, 2011; Hübner et al., 2010; Ong et al., 2017; Soares et al., 2019; Ulrich et al., 2015; White et al., 2011). Most relevant to our research is a study by Aschenbrenner (2016; Experiment 2) in which he applied the standard diffusion model to a Simon task to examine the roots of the CSE. He could locate the effect in both drift rate and non-decision time. In contrast to our study, no reward manipulation was used and only the CSE, not the PCE, was examined. To the best of our knowledge, the diffusion model has not yet been applied for an examination of effects of reward prospect on context-varying congruency effects like the PCE or CSE.

We conducted two experiments which focused on the PCE (Experiment 1) and the CSE (Experiment 2), respectively. In both experiments, we used a number Simon task, manipulated the reward prospect (none vs. performance-contingent vs. non-contingent) and inspected both behavioral variables and diffusion model parameters. We expected to find an increased PCE and probably CSE in terms of behavioral variables (mean RT and error rate) in the performance-contingent reward condition and a reduced PCE and CSE in the non-contingent reward condition. Further, we were interested in which parameter(s) of the diffusion model such an interaction effect is possibly captured.

## Experiment 1

In Experiment 1, we investigated the influence of performance (non-)contingent reward on context processing in a conflict task with a proportion congruency manipulation. Accordingly, the distracting context information was predictive for the probability of the upcoming congruency. Therefore, we expected to find a similar dissociation to that found in previous studies using the AX-CPT (Fröber & Dreisbach, 2014, 2016a), with more usage of context information under performance-contingent reward prospect and less reliance on context information under non-contingent reward prospect.

### Methods

#### Participants

We determined the minimum sample size by means of a power analysis using MorePower 6.0.4 (Campbell & Thompson, 2012). Alpha was set to 5%, intended power to 90%, and the effect size was taken from the three-way interaction in the global reward effects analysis from Fröber and Dreisbach (2016a, Experiment 1). With the repeated-measures design of the present study, the power analysis determined a minimum sample size of 30 participants. Because it is not known whether results from the AX-CPT studies are applicable to reward prospect effects on control adaptations in conflict tasks, and with respect to the unconclusive results in the conflict literature so far, we decided to recruit a substantially larger sample size. Thus, a total of 83 undergraduate students from the University of Regensburg participated in Experiment 1 for partial course credit. Of these, 50 were tested in the lab, and 33 online. All participants provided signed informed consent before taking part in the experiment. Participants were on average 22.30 years old (*SD* = 4.55, range: 18–51 years). The percentage of female participants was 87.95%.

#### Apparatus, stimuli, and task

Data collection started in the lab on a PC with E-Prime 2.0 (Psychology Software Tools, USA). All stimuli were presented on a 17-in. monitor (30 cm × 37.5 cm; resolution 1,280 × 1,024 pixels; refresh rate 75 Hz) located approximately 60 cm from the participant. Responses were collected with a QWERTZ-keyboard with the Y and M key serving as left and right response key, respectively. Due to the COVID-19 pandemic, data collection was changed to an online experiment for the last 33 participants. Consequently, no specific information can be given about the apparatus. Participants were asked to use a laptop or PC with a QUERTZ keyboard, and to close all irrelevant programs or additional browser tabs during the experiment. The online experiment was programmed equivalent to the E-Prime lab version in lab.js (Henninger et al., 2022) and data collection was done using OpenLab (Shevchenko, 2022).

Target stimuli were single digits from 1 to 9, excluding 5 (font: Calibri; size: 24 pt) presented in black on a white background. The digits were presented 10% left or right from a central fixation cross. Participants were instructed to press the left response key for digits < 5 and the right response key for digits > 5, using a fixed response mapping with intuitive compatibility (Dehaene et al., 1993). Congruent trials comprised digits < 5 on the left or digits > 5 on the right side of the screen, conversely incongruent trials comprised digits < 5 on the right or digits > 5 on the left side.

#### Procedure

The experiment lasted about 40 min and comprised three phases: practice, baseline, and reward. The experiment started with basic instructions on the task followed by a practice block of 32 trials, consisting of all eight target stimuli, once in a congruent and once in an incongruent mode, presented twice in random order. The following baseline phase established the proportion congruency manipulation and comprised six short blocks of 48 trials each, of which there were three mostly congruent and three mostly incongruent blocks, in random succession. Participants were instructed to respond as fast and as accurately as possible. The proportion congruency manipulation was only implemented in the first 32 trials of a block, with 75% of these trials being mostly congruent or incongruent. The remaining 16 trials had an equal proportion of congruent and incongruent trials to establish a neutral transition phase between blocks. Each block was followed by a short break with a fixed duration of 8 s. The baseline phase served to calculate individual response criteria that were used in the performance-contingent condition of the subsequent reward phase. Separately for each combination of proportion congruency (levels: mostly congruent vs. mostly incongruent) and congruency N (i.e., the congruency in the current trial; levels: congruent vs. incongruent) the fasted third of the correct RTs was determined as the response criterion.

At the beginning of the reward phase, participants were informed that they would enter a social competition for the rest of the experiment. It was explained that after the end of data collection the three participants with the best scores would win a special prize. They were furthermore told that at the beginning of each block a random generator (symbolized with a picture of two dice) would determine whether participants could earn points (performance-contingent reward), were given points as a gift (non-contingent reward), or had no opportunity of getting rewards (neutral control) in the upcoming block. Reward conditions were not actually determined randomly, instead each type of reward (contingent vs. non-contingent vs. neutral) was combined twice, with each proportion congruency condition (mostly congruent vs. mostly incongruent) resulting in 12 blocks in total. In a performance-contingent reward block, 7 points could be earned for each correct response faster than the individual response threshold. In a non-contingent reward block, each response irrespective of performance – that is, even errors and very slow responses – was rewarded with 7 points. Similar to the baseline phase, each block was 48 trials long with a proportion congruency manipulation in the first 32 trials only. Thus, in the reward phase, participants worked on a total of 12 × 48 = 576 trials. Between blocks the random generator was shown for 4 s, followed by the instruction on the given reward condition in the next block for another 4 s. After six blocks participants could take a longer break of self-determined duration. In this break, they got feedback on the total sum of points already gained during the experiment.

A single trial in the experiment started with the presentation of a fixation cross. After 500 ms a target stimulus appeared left or right from the fixation cross and remained on-screen until response. The trial ended with a feedback message shown for 1,000 ms. In practice, baseline and neutral trials from the reward phase, the feedback message informed about accuracy only (“Richtig” [“Correct”] or “Falsch” [“Wrong”]). In a performance-contingent block, the feedback message read “Richtig. + 7 Punkte” [“Correct. + 7 points”] after correct and fast enough responses, “Zu langsam. Keine Punkte” [“Too slow. No points”] after correct but too slow responses, or “Falsch. Keine Punkte” [“Wrong. No points”] after erroneous responses. In a non-contingent block, the feedback message read “Richtig. + 7 Punkte” [“Correct. + 7 points”] after correct responses and “Falsch. + 7 Punkte” [“Wrong. + 7 points”] after erroneous responses.

#### Diffusion modeling: Parameter estimation procedure

We applied the diffusion model to the data of the reward phase. For parameter estimation, we used the optimization criterion Maximum Likelihood implemented in the program *fast-dm-30* (Voss et al., 2015; Voss & Voss, 2007, 2008). Parameters were estimated separately for each participant. The two thresholds of the model were associated with correct (upper threshold) and erroneous responses (lower threshold, see also Fig. 1). Accordingly, the starting point was set to the center between the two thresholds (i.e., *z*_*r*_ = .5; participants cannot have a bias for either correct or erroneous responses). Drift rate, threshold separation, and non-decision time were allowed to vary by Type of Reward (none vs. performance-contingent vs. non-contingent) and Proportion Congruency (mostly congruent vs. mostly incongruent). Drift rate and non-decision time were further allowed to vary by Congruency N (congruent vs. incongruent), that is, the congruency of the stimulus in the current trial. According to the definition of the diffusion model, the threshold separation is set *before* the beginning of the trial. Therefore, this parameter cannot vary by properties of the current trial. The intertrial variabilities of starting point and drift rate were fixed at the value 0 to improve estimation of the main diffusion model parameters (Lerche & Voss, 2016; see also Böhm et al., 2018; van Ravenzwaaij et al., 2017). In contrast, the intertrial variability of non-decision time, which is usually estimated better than the other two intertrial variabilities and can improve parameter estimation in particular when Maximum Likelihood is used (Lerche & Voss, 2016), was estimated (one estimate across all conditions).

### Results

We used the statistical program *R* (R Core Team, 2021) for data processing and the analyses. All significance tests were conducted as two-sided tests and were based on a significance level of 5%.

#### Data preprocessing

Practice trials, the first trial of each block and the last 16 trials of each block (i.e., the neutral transition phase with an equal proportion of congruent and incongruent trials) were removed. Accordingly, the remaining data of each participant consisted of 372 trials. Next, all trials with negative RTs were removed (none of the trials in the baseline phase and on average 0.04% of the trials in the reward phase)^3^. In addition, we identified trials as outliers that deviated more than three standard deviations from the mean of the respective cell (i.e., the combination of the factors Type of Reward, Proportion Congruency, and Congruency N). This exclusion procedure resulted in the removal of 1.61% and 1.27% of the trials in the baseline and reward phase, respectively. Finally, with regard to the participants, we identified outliers based on the behavioral variables mean RT of correct responses and error rate in the baseline phase. Specifically, participants who were more than 3 interquartile ranges outside of the third quartile were removed (three participants; see Tukey, 1977). The total trial number for the remaining participants was between 363 and 371 trials and the trial number per condition varied between 13 and 48.^4^

#### Baseline phase – mean RTs and error rates

**Fig. 2:**
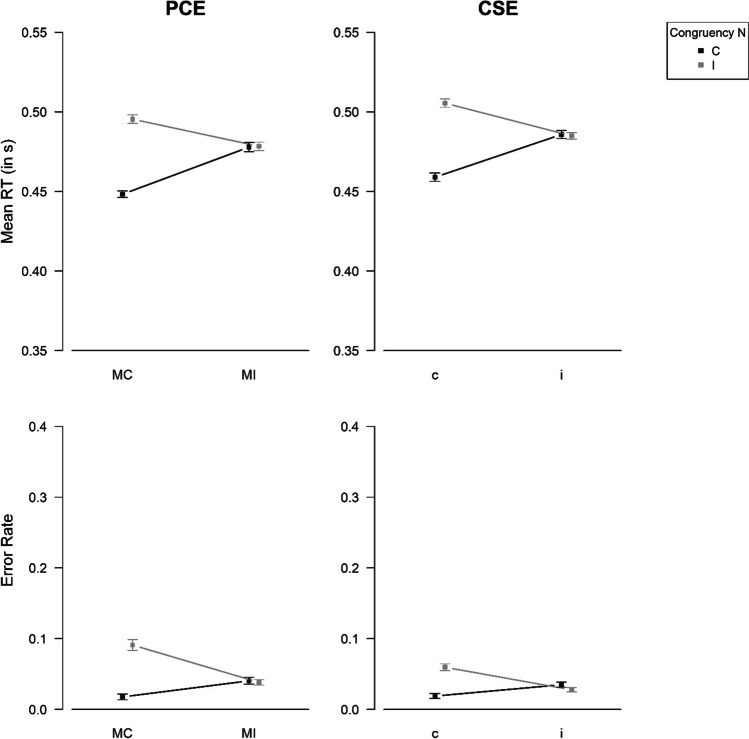
Effects of Congruency N and Proportion Congruency (Exp. 1)/Congruency N-1 (Exp. 2) on the behavioral variables mean response time (RT) of correct responses and error rate in the baseline phase. *Note.* PCE = proportion congruency effect, CSE = congruency sequence effect, MC = mostly congruent, MI = mostly incongruent, C = congruent (trial N), I = incongruent (trial N), c = congruent (trial N-1), i = incongruent (trial N-1). The figure shows means and errors bars, which are standard errors according to the procedure for within-subject designs proposed by Cosineau and O’Brien (2012)

We were mostly interested in the data of the reward phase. However, preceding the analysis of this data, we examined the data of the baseline phase to check whether the proportion congruency manipulation worked as intended. To this aim, we conducted two 2 (Proportion Congruency) × 2 (Congruency N) repeated-measures ANOVAs with the variables error rate and mean RT of correct responses (for simplicity, in the following termed “mean RT”) as dependent measure, respectively. A summary of all effects is given in Table 1. Most importantly, the Proportion Congruency × Congruency N interaction effects were significant (error rate: *F*[1, 79] = 43.75, *p* < .001, *η*_p_^2^ = 0.36; mean RT: *F*[1, 79] = 124.11, *p* < .001, *η*_p_^2^ = 0.61). Figure 2 (left column) illustrates the pattern of results. Simple effect analyses showed a large congruency effect for blocks with mostly congruent trials (*t*-test for error rate: *t*[79] = -7.50, *p* < .001, *d*_*z*_ = -0.84; *t*-test for mean RT: *t*[79] = -15.26, *p* < .001, *d*_*z*_ = -1.71), whereas there was no significant congruency effect in blocks with mostly incongruent trials (*t*-test for error rate: *t*[79] = 0.36, *p* = .720, *d*_*z*_ = 0.04; *t*-test for mean RT: *t*[79] = -0.14, *p* = .892, *d*_*z*_ = -0.02).^5^

**Table 1:** Results of repeated-measures ANOVAs for the Baseline Phase of Experiment 1

	Error rate		Mean RT	
	*F*	𝜂_p_^2^	*F*	𝜂_p_^2^
Congruency N	36.08***	0.31	71.29***	0.47
Proportion Congruency	13.25***	0.14	5.35*	0.06
Proportion Congruency × Congruency N	43.75***	0.36	124.11***	0.61

* *p* < .05. ** *p* < .01. *** *p* < .001; *RT* response time

#### Reward phase – mean RTs, error rates, and diffusion model parameters

**Fig. 3:**
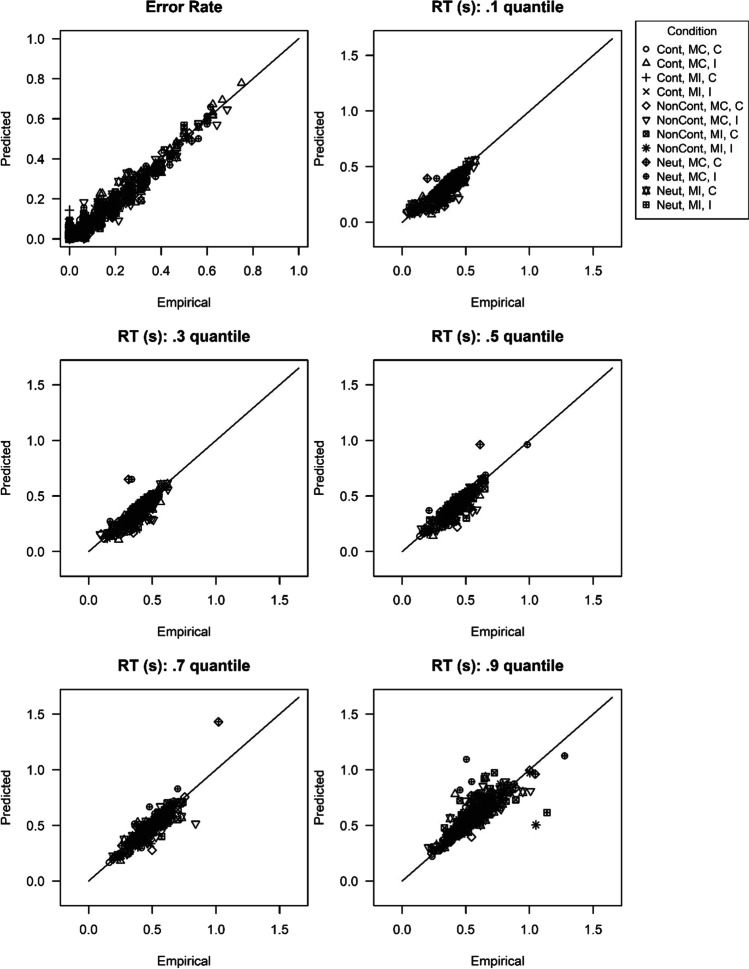
Scatterplots for comparison of empirical and estimated statistics in Experiment 1. *Note.* The different symbols indicate the different cells of the experimental design (Cont = performance-contingent, Neut = neutral, NonCont = non-contingent, MC = mostly congruent, MI = mostly incongruent, C = congruent, I = incongruent). As they exceed the scales of a response time (RT) quantile plot, three data points (one in the .7 RT quantile plot and two in the .9 RT quantile plot) are not depicted

Regarding the data of the reward phase, we examined not only the behavioral variables error rate and mean RT, but additionally the DDM parameters drift rate, non-decision time and threshold separation to differentiate between possible mechanisms involved. To make sure that we could analyze the estimated parameters of the diffusion model, we first examined the model fit. Figure 3 shows scatterplots that illustrate the fit of the diffusion model to characteristics of the empirical data (more specifically, the error rate and several RT quantiles). Each symbol indicates one participant in one of the 3 (Type of Reward) × 2 (Proportion Congruency) × 2 (Congruency N) = 12 cells. Most importantly, almost all symbols are close to the diagonal line, with this line indicating perfect model fit, i.e., a perfect match of the empirically observed statistic and the statistic computed based on the estimated parameters. Thus, the fit of the diffusion model to the data was convincing.

**Fig. 4:**
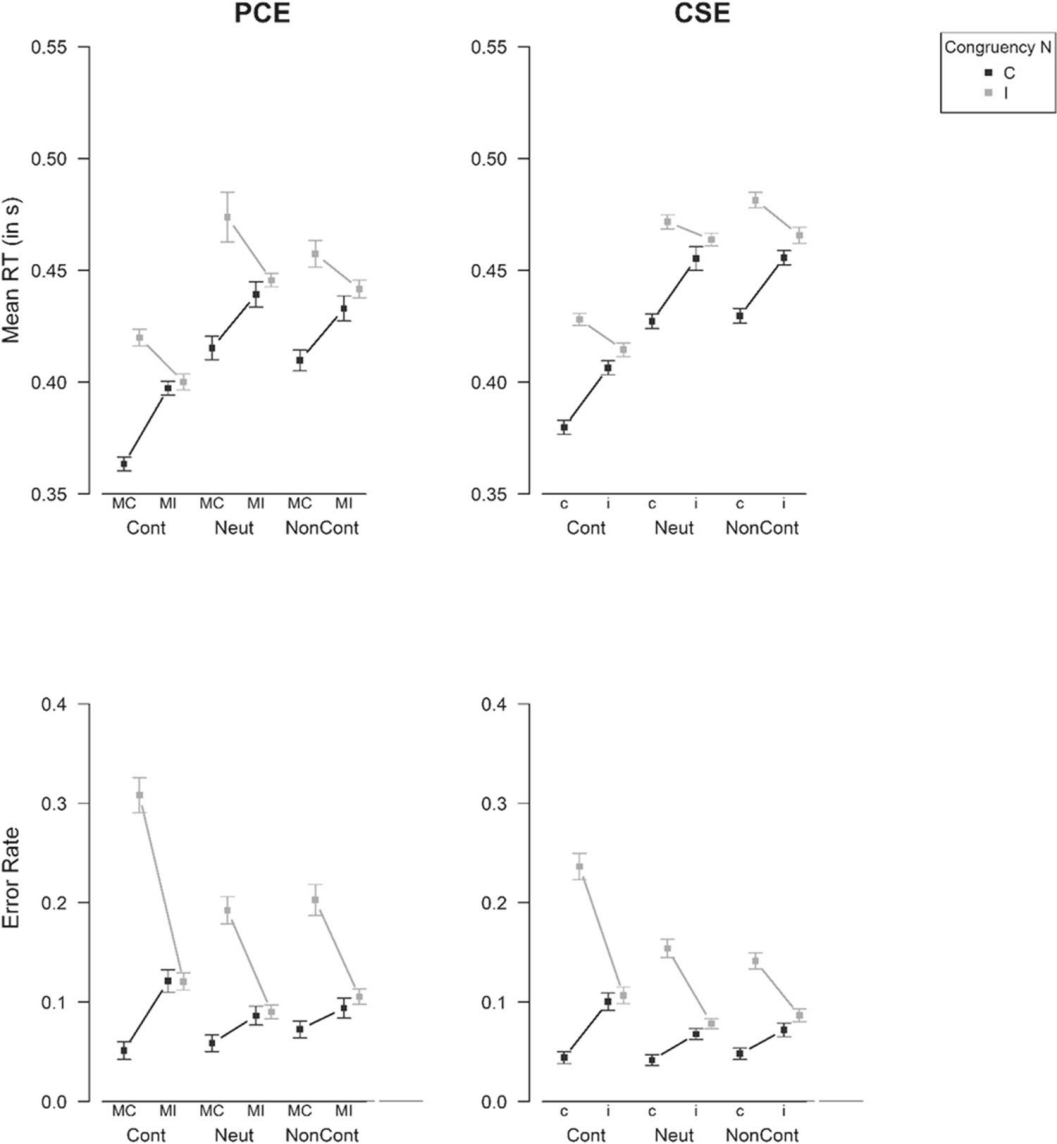
Effects of Congruency N and Proportion Congruency (Exp. 1)/Congruency N-1 (Exp. 2) on the behavioral variables mean response time (RT) of correct responses and error rate in the reward phase. *Note.* PCE = proportion congruency effect, CSE = congruency sequence effect, Cont = performance-contingent, Neut = neutral, NonCont = non-contingent, MC = mostly congruent, MI = mostly incongruent, C = congruent (trial N), I = incongruent (trial N), c = congruent (trial N-1), i = incongruent (trial N-1). The figure shows means and errors bars, which are standard errors according to the procedure for within-subject designs proposed by Cosineau and O’Brien (2012)

**Fig. 5:**
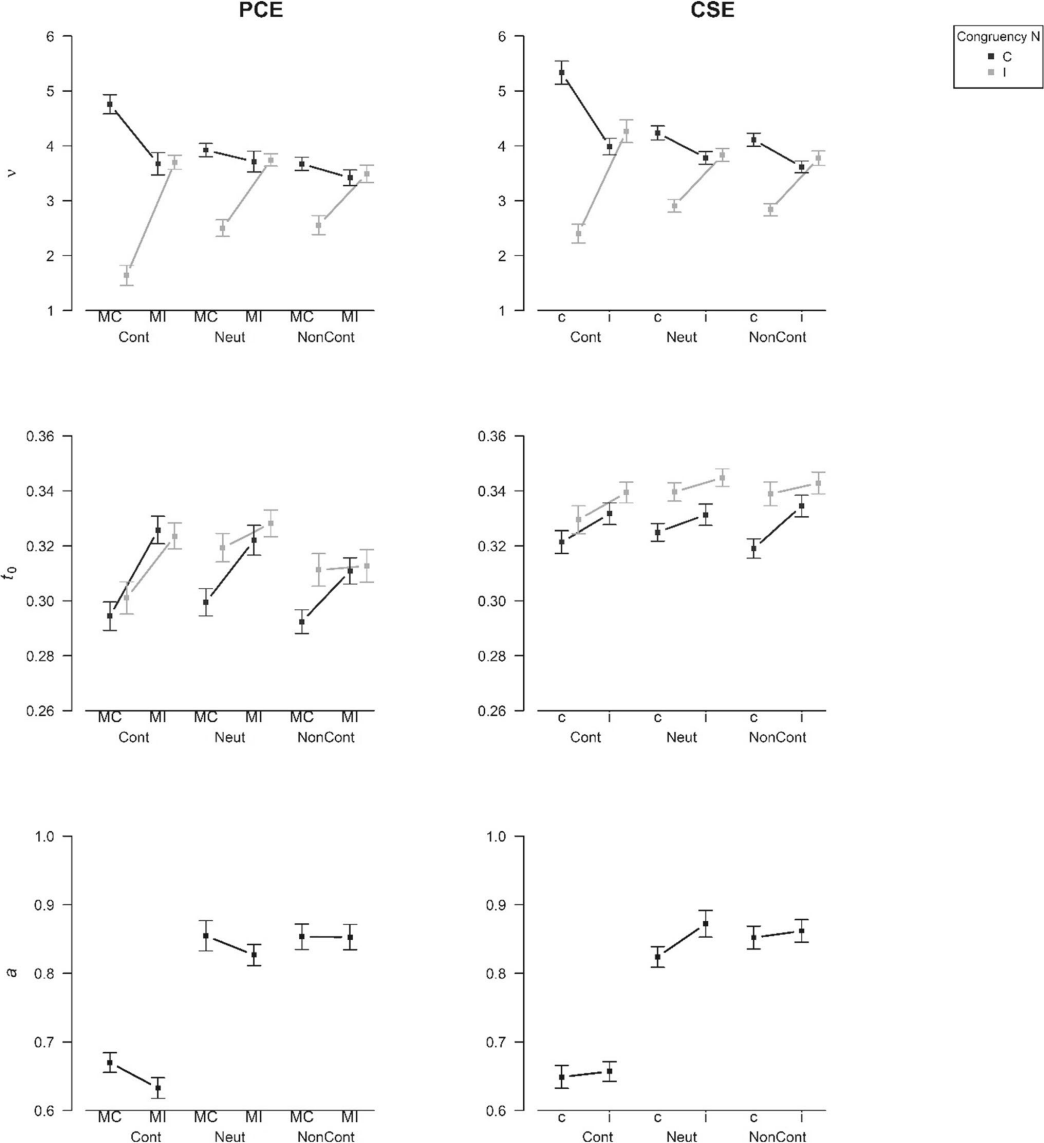
**E**ffects of Congruency N and Proportion Congruency (Exp. 1)/Congruency N-1 (Exp. 2) on the diffusion model parameters in the reward phase. *Note.* PCE = proportion congruency effect, CSE = congruency sequence effect, Cont = performance-contingent, Neut = neutral, NonCont = non-contingent, MC = mostly congruent, MI = mostly incongruent, C = congruent (trial N), I = incongruent (trial N), c = congruent (trial N-1), i = incongruent (trial N-1). The figure shows means and errors bars, which are standard errors according to the procedure for within-subject designs proposed by Cosineau and O’Brien (2012)

For the variables mean RT, error rate, drift rate, and non-decision time, we conducted a 3 (Type of Reward) × 2 (Proportion Congruency) × 2 (Congruency N) repeated-measures ANOVA, respectively. For threshold separation a 3 (Type of Reward) × 2 (Proportion Congruency) repeated-measures ANOVA was computed. Here, we will focus on the effects that our research questions center on. However, a summary of all main and interaction effects can be found in Table 2. For a graphical illustration of the effects, see Fig. 4 (behavioral variables) and Fig. 5 (diffusion model parameters) (left columns for Experiment 1). Like in the baseline condition, we observed a 2 (Proportion Congruency) × 2 (Congruency N) interaction effect that was apparent in error rate (*F*[1, 79] = 190.74, *p* < .001, *η*_p_^2^ = 0.71) and mean RT (*F*[1, 79] = 176.24, *p* < .001, *η*_p_^2^ = 0.69). This interaction effect also showed up in the diffusion model parameters drift rate (*F*[1, 79] = 106.32, *p* < .001, *η*_p_^2^ = 0.57) and non-decision time (*F*[1, 79] = 15.12, *p* < .001, *η*_p_^2^ = 0.16). Notably, the two-way interaction in error rate was moderated by the Type of Reward, *F*(1.95, 153.80) = 20.48, *p* < .001, *η*_p_^2^ = 0.21. As can be seen in Fig. 4, in the performance-contingent reward condition, the two-way interaction was larger than in the other two conditions. Separately for each reward condition, we conducted Proportion Congruency × Congruency N repeated-measures ANOVAs. There was a significant two-way interaction for error rate in all three reward conditions. However, the interaction effect was larger for the performance-contingent reward condition (*F*[1, 79] = 162.02, *p* < .001, *η*_p_^2^ = 0.67) than for the neutral (*F*[1, 79] = 56.42, *p* < .001, *η*_p_^2^ = 0.42) and non-contingent reward condition (*F*[1, 79] = 44.01, *p* < .001, *η*_p_^2^ = 0.36). Interestingly, a three-way interaction effect also showed up in drift rate, *F*(1.94, 152.95) = 16.54, *p* < .001, *η*_p_^2^ = 0.17. The pattern was very similar to the one for error rate (see Fig. 5). Again, in all three reward conditions, a significant Proportion Congruency × Congruency N interaction emerged, with a larger effect size in the performance-contingent reward condition (*F*[1, 79] = 123.38, *p* < .001, *η*_p_^2^ = 0.61) than in the neutral reward condition (*F*[1, 79] = 26.18, *p* < .001, *η*_p_^2^ = 0.25) or the non-contingent reward condition (*F*[1, 79] = 17.60, *p* < .001, *η*_p_^2^ = 0.18). In mean RT (*F*[1.74, 137.21] = 2.49, *p* = .094, *η*_p_^2^ = 0.03) and non-decision time (*F*[1.82, 144.01] = 0.70, *p* = .484, *η*_p_^2^ = 0.01), on the other hand, no significant three-way interactions emerged.^6^^,^^7^

**Table 2:** Results of repeated-measures ANOVAs for Reward Phase of Experiment 1

	Error rate		Mean RT		𝜈		*t*_*0*_		*a*	
	*F*	𝜂_p_^2^	*F*	𝜂_p_^2^	*F*	𝜂_p_^2^	*F*	𝜂_p_^2^	*F*	𝜂_p_^2^
Congruency N	114.87***	0.59	112.61***	0.59	78.52***	0.50	27.77***	0.26		
Proportion Congruency	68.07***	0.46	0.66	0.01	28.56***	0.27	34.59***	0.30	3.19	0.04
Type of Reward	7.96***	0.09	37.11***	0.32	0.93	0.01	1.24	0.02	65.67***	0.45
Proportion Congruency × Congruency N	190.74***	0.71	176.24***	0.69	106.32***	0.57	15.12***	0.16		
Type of Reward × Proportion Congruency	2.35	0.03	0.70	0.01	0.57	0.01	2.98	0.04	0.69	0.01
Type of Reward × Congruency N	15.35***	0.16	0.39	0.00	14.37***	0.15	4.20*	0.05		
Type of Reward × Proportion Congruency × Congruency N	20.48***	0.21	2.49	0.03	16.54***	0.17	0.70	0.01		

*Note*. * *p* < .05. ** *p* < .01. *** *p* < .001. *RT* response time

In threshold separation, we found a significant main effect of Type of Reward (*F*[1.89, 149.08] = 65.67, *p* < .001, *η*_p_^2^ = 0.45). Post hoc tests (based on Tukey’s procedure) revealed that threshold separation was significantly smaller in the performance-contingent compared to the neutral (*p* < .001) and the non-contingent reward condition (*p* < .001), whereas there was no significant difference between the neutral and the non-contingent condition (*p* = .840). Thus, participants were less cautious in the condition that put a focus on both accuracy and RT. The effect of Type of Reward was not moderated by Proportion Congruency (*F*[1.87, 147.40] = 0.69, *p* = .492, *η*_p_^2^ = 0.01).^8^

### Discussion

We found a reliable PCE in Experiment 1, in both the non-rewarded baseline and the reward phase and in both error rate and mean RT. Within the reward phase, the PCE in the error rate was furthermore modulated by the reward condition, with an increased PCE under contingent reward prospect. Theoretically important, this three-way interaction was due to an increased congruency effect in mostly congruent blocks while the congruency effect in mostly incongruent blocks was equally reduced in all three reward conditions. Results from the diffusion model analysis furthermore revealed a significant PCE in the drift rate and non-decision time parameter, but only in the drift rate was a moderation by the reward condition found that was similar to the effect seen in the error rates. A significant main effect of Type of Reward was found in the threshold separation parameter, which, however, did not interact with the proportion congruency manipulation.

Taken together, participants seemed to adapt their response criterion under performance-contingent reward prospect as suggested by the reduced threshold separation parameter. In addition, the modulation of the PCE in error rate and drift rate with an increased congruency effect in mostly congruent blocks fits with the expected increase in the usage of context information. When congruent trials were frequent, participants in the performance-contingent reward prospect seemed to make increased use of the mostly congruent distractor information. This confirms previous findings found with the AX-CPT paradigm (Chiew & Braver, 2013, 2014; Fröber & Dreisbach, 2014, 2016a; Hefer & Dreisbach, 2016, 2017, 2020a). Interestingly, control adaptions in mostly incongruent blocks were not moderated by reward condition, and no differences in control adaptations between the non-contingent reward prospect and the neutral control condition were found. We will come back to these issues in the *General discussion*.

## Experiment 2

Experiment 2 focused on the CSE. That is, in contrast to Experiment 1, there was an equal proportion of congruent and incongruent trials in each block. Accordingly, the distracting context information was not predictive of the upcoming congruency condition anymore, and thus less similar to the predictive context information in an AX-CPT. Results will show whether participants nonetheless show signs of increased context processing under performance-contingent reward prospect.

### Methods

#### Participants

Power analyses with MorePower based on the significant three-way interactions in analyses of error rate and drift rate in Experiment 1 suggested a minimum sample size of 26 and 34 participants, respectively. Because context information in Experiment 2 was no longer predictive of the upcoming congruency, the generalizability of the results was unclear, so that a substantially larger sample was recruited. Another sample of 91 undergraduate students from the University of Regensburg participated in Experiment 2 for partial course credit. Of these, 60 were tested in the lab, and 31 online. All participants provided signed informed consent before taking part in the experiment. Participants were on average 22.87 years old (*SD* = 4.50, range: 18–47 years). The percentage of female participants was 84.62%.

#### Apparatus, stimuli, and task

Apparatus, stimuli, and task were the same as in Experiment 1. Apparatus could again only be controlled for data collection in the lab.

#### Procedure

Analogous to Experiment 1, Experiment 2 comprised three phases and lasted about 35 min in total. The practice phase was the same as in Experiment 1. The baseline phase consisted of a single block of 128 trials with an equal proportion of congruent and incongruent trials in a pseudorandomized order to present all four sequences (congruent-congruent [cC], incongruent-congruent [iC], congruent-incongruent [cI], and incongruent-incongruent [iI]) approximately equally distributed. Again, the baseline phase served to calculate individual response criteria, separately for each congruency sequence, for the performance-contingent condition in the subsequent reward phase.

The reward phase of Experiment 2 used the same reward conditions and instructions as in Experiment 1. Again, the reward condition was supposedly determined by a random generator and announced for a short block of 32 trials. These blocks had a pseudo-randomized order similar to the baseline phase with an approximately equal distribution of congruency sequences. The reward phase comprised 12 blocks in total, again separated by a break of self-determined duration after six blocks. Thus, in the reward phase, participants worked on a total of 12 × 32 = 384 trials. With the total sum of points received during the reward phase, participants again entered a social competition across all participants.

The procedure of a single trial was identical to Experiment 1.

#### Diffusion modeling: Parameter estimation procedure

The parameter estimation procedure was basically the same as in Experiment 1. In contrast to Experiment 1, in Experiment 2 the factor Proportion Congruency was replaced by the factor Congruency N-1 (congruent vs. incongruent), i.e., the congruency of the stimulus in the preceding trial. Accordingly, drift rate, threshold separation, and non-decision time were allowed to vary by Type of Reward (none vs. performance-contingent vs. non-contingent) and Congruency N-1. Drift rate and non-decision time were further allowed to vary by Congruency N (congruent vs. incongruent).

### Results

To examine the data of Experiment 2, we conducted repeated-measures ANOVAs like for the data of Experiment 1, with the only difference being that the factor Proportion Congruency was replaced by the factor Congruency N-1.

#### Data preprocessing

Practice trials and the first trial of each block were removed. Accordingly, like in Experiment 1, the remaining data of each participant consisted of 372 trials. Next, all trials with negative RTs were removed (none of the trials in the baseline phase and on average 0.14% of the trials in the reward phase). In addition, we identified trials as outliers that deviated more than three standard deviations from the mean of the respective cell (i.e., combination of the factors Type of Reward, Congruency N-1, and Congruency N). This exclusion procedure resulted in the removal of 1.49% and 1.46% of the trials in the baseline and reward phase, respectively. Based on the outlier exclusion criterion also applied in Experiment 1 one participant was identified as an outlier. Two further participants were excluded because the diffusion model analyses resulted in a penalty message.^9^ The total trial number for the remaining participants was between 326 and 371 trials and the trial number per condition varied between 25 and 36.

#### Baseline phase – mean RTs and error rates

A summary of all effects of the 2 (Congruency N-1) × 2 (Congruency N) repeated-measures ANOVAs is given in Table 3. Most importantly, the Congruency N-1 × Congruency N interaction effects were significant (error rate: *F*[1, 87] = 37.34, *p* < .001, *η*_p_^2^ = 0.30; mean RT: *F*[1, 87] = 127.28, *p* < .001, *η*_p_^2^ = 0.59). Figure 2 (right column) illustrates the pattern of results. Simple effect analyses showed substantial congruency effects for preceding congruent trials (*t*-test for error rate: *t*[87] = -6.38, *p* < .001, *d*_*z*_ = -0.68; *t*-test for mean RT: *t*[87] = -11.39, *p* < .001, *d*_*z*_ = -1.21), whereas there was no congruency effect if the preceding trial was incongruent (*t*-test for error rate: *t*[87] = 1.54, *p* = .128, *d*_*z*_ = 0.16; *t*-test for mean RT: *t*[87] = 0.25, *p* = .805, *d*_*z*_ = 0.03).^10^

**Table 3:** Results of repeated-measures ANOVAs for Baseline Phase of Experiment 2

	Error rate		Mean RT	
	*F*	𝜂_p_^2^	*F*	𝜂_p_^2^
Congruency N	17.36***	0.17	50.89***	0.37
Congruency N-1	6.14*	0.07	2.86	0.03
Congruency N-1 × Congruency N	37.34***	0.30	127.28***	0.59

* *p* < .05. ** *p* < .01. *** *p* < .001. *RT* response time

#### Reward phase – mean RTs, error rates, and diffusion model parameters

**Fig. 6:**
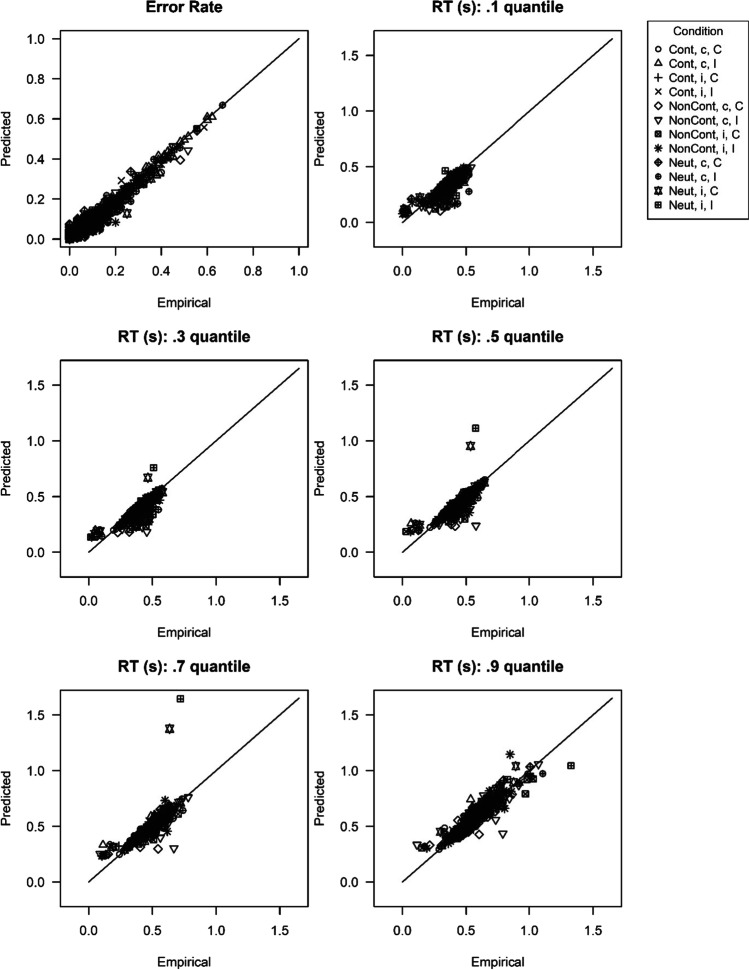
Scatterplots for comparison of empirical and estimated statistics in Experiment 2. *Note.* The different symbols indicate the different cells of the experimental design (Cont = performance-contingent, Neut = neutral, NonCont = non-contingent, MC = mostly congruent, MI = mostly incongruent, C = congruent, I = incongruent). As they exceed the scales of the .9 response time (RT) quantile plot, two data points are not depicted

Like in Experiment 1, the scatterplots (see Fig. 6) indicate a good fit of the diffusion model. For the variables mean RT, error rate, drift rate, and non-decision time, we conducted a 3 (Type of Reward) × 2 (Congruency N-1) × 2 (Congruency N) repeated-measures ANOVA, respectively. For threshold separation a 3 (Type of Reward) × 2 (Congruency N-1) repeated-measures ANOVA was computed. A summary of all main and interaction effects can be found in Table 4. For a graphical illustration of the effects, see the right columns of Fig. 4 (behavioral variables) and Fig. 5 (diffusion model parameters). Interestingly, the results were very similar to the findings of Experiment 1. Like in the baseline condition and like in Experiment 1, we observed a 2 (Congruency N-1) × 2 (Congruency N) interaction effect that was apparent in error rate (*F*[1, 87] = 202.32, *p* < .001, *η*_p_^2^ = 0.70) and mean RT (*F*[1, 87] = 130.00, *p* < .001, *η*_p_^2^ = 0.60). This interaction effect also showed up in the diffusion model parameter drift rate (*F*[1, 87] = 152.52, *p* < .001, *η*_p_^2^ = 0.64) and a marginally significant effect was found for non-decision time (*F*[1, 87] = 2.90, *p* = .092, *η*_p_^2^ = 0.03). Again like in Experiment 1, the two-way interaction in error rate was moderated by Type of Reward, *F*(1.70, 147.85) = 24.03, *p* < .001, *η*_p_^2^ = 0.22. As can be seen in Fig. 4, in the performance-contingent reward condition, the two-way interaction was larger than in the other two conditions. Separately for each reward condition, we conducted Congruency N-1 × Congruency N repeated-measures ANOVAs. There was a significant two-way interaction for error rate in all three reward conditions. However, the interaction effect was larger for the performance-contingent reward condition (*F*[1, 87] = 140.93, *p* < .001, *η*_p_^2^ = 0.62) than for the neutral (*F*[1, 87] = 74.31, *p* < .001, *η*_p_^2^ = 0.46) and non-contingent reward condition (*F*[1, 87] = 61.34, *p* < .001, *η*_p_^2^ = 0.41). Like in Experiment 1, the three-way interaction was also found for drift rate, *F*(1.75, 152.60) = 18.52, *p* < .001, *η*_p_^2^ = 0.18 (see also Fig. 5). In all three reward conditions, a significant Congruency N-1 × Congruency N interaction emerged, with a larger effect size in the performance-contingent reward condition (*F*[1, 87] = 99.69, *p* < .001, *η*_p_^2^ = 0.53) than in the neutral reward condition (*F*[1, 87] = 38.39, *p* < .001, *η*_p_^2^ = 0.31) or the non-contingent reward condition (*F*[1, 87] = 47.30, *p* < .001, *η*_p_^2^ = 0.35). In mean RT (*F*[1.96, 170.70] = 0.66, *p* = .513, *η*_p_^2^ = 0.01) and non-decision time (*F*[1.76, 153.29] = 1.89, *p* = .159, *η*_p_^2^ = 0.02); on the other hand, no significant three-way interactions emerged.^11^,^12^

**Table 4:** Results of repeated-measures ANOVAs for Reward Phase of Experiment 2

	Error rate		Mean RT		𝜈		*t*_*0*_		*a*	
	*F*	𝜂_p_^2^	*F*	𝜂_p_^2^	*F*	𝜂_p_^2^	*F*	𝜂_p_^2^	*F*	𝜂_p_^2^
Congruency N	101.95***	0.54	173.06***	0.67	47.89***	0.36	86.74***	0.50		
Congruency N-1	45.50***	0.34	23.79***	0.21	12.34***	0.12	27.30***	0.24	2.59	0.03
Type of Reward	19.01***	0.18	93.52***	0.52	6.31**	0.07	0.40	0.00	78.85***	0.48
Congruency N-1 × Congruency N	202.32***	0.70	130.00***	0.60	152.52***	0.64	2.90	0.03		
Type of Reward × Congruency N-1	3.28*	0.04	1.17	0.01	0.02	0.00	0.74	0.01	1.29	0.01
Type of Reward × Congruency N	12.43***	0.12	0.88	0.01	10.36***	0.11	2.94	0.03		
Type of Reward × Congruency N-1 × Congruency N	24.03***	0.22	0.66	0.01	18.52***	0.18	1.89	0.02		

* *p* < .05. ** *p* < .01. *** *p* < .001. *RT* response time

Like in Experiment 1, we found a significant main effect of Type of Reward on threshold separation (*F*[1.94, 169.08] = 78.85, *p* < .001, *η*_p_^2^ = 0.48). Post hoc tests (based on Tukey’s procedure) revealed that threshold separation was significantly smaller in the performance-contingent compared to the neutral (*p* < .001) and the non-contingent reward condition (*p* < .001), whereas there was no significant difference between the neutral and the non-contingent condition (*p* = .861). Thus, like in Experiment 1, participants were less cautious in the condition that put a focus on both accuracy and RT. The effect of Type of Reward was not moderated by Congruency N-1 (*F*[1.99, 173.24] = 1.29, *p* = .278, *η*_p_^2^ = 0.01).^13^

### Discussion

Across both phases and both behavioral variables mean RT and error rate, we found a reliable CSE. In contrast to Experiment 1, congruency in Experiment 2 was no longer predictive of the upcoming congruency. Nonetheless, we found a very similar pattern of results: The reliable CSE effect was further modulated by the reward condition in both error rate and drift rate. More precisely and similar to the increased congruency effect in mostly congruent blocks in Experiment 1, the congruency effect after congruent trials was further increased under performance-contingent reward prospect. Furthermore, the (independent) main effect of Type of Reward on threshold separation replicated across experiments. Taken together, even in an unpredictable context, performance-contingent reward seemed to increase the use of congruent distractor information, while control adaptations following incongruent trials were again not further modulated by reward condition.

## General discussion

The present study was aimed to investigate the effect of performance (non-)contingent reward prospect on context processing in a conflict task, for which the existing literature is characterized by heterogeneous results and diverse methods. Context processing was investigated in terms of two control adaptation phenomena, namely the PCE (Experiment 1) and the CSE (Experiment 2). In addition to the behavioral measures error rate and mean RT, we analyzed our data with the DDM. In both experiments, we found a three-way interaction of Type of Reward, Congruency N, and Proportion Congruency (Experiment 1)/Congruency N-1 (Experiment 2) in the error rate that was mirrored by results in the DDM parameter drift rate. This interaction was due to an increased congruency effect specifically under performance-contingent reward prospect in mostly congruent blocks (Experiment 1) or after congruent trials (Experiment 2). That is, performance-contingent reward prospect seemed to further boost the use of task-irrelevant context information compared to non-contingent reward prospect and a control condition without reward. Interestingly, the adaptation to (mostly) incongruent trials did not differ between reward conditions. In all three conditions, the congruency effect in mostly incongruent blocks or following incongruent trials was equally reduced to a non-significant difference. These results suggest a functional role of both incongruent and congruent trials in control adaptations, while only the adaptation to congruent trials seems responsive to the motivational influence by performance-contingent reward prospect.

### Performance-contingent reward prospect increases the usage of congruent distractor information

Performance-contingent reward prospect motivated for improved performance in terms of increased response speed compared to non-contingent reward prospect and the neutral control condition without reward. Furthermore, with respect to the PCE (Experiment 1) and CSE (Experiment 2), an increased congruency effect with less errors in congruent trials and more errors in incongruent trials was found in mostly congruent blocks or after congruent trials. This is in line with our assumption of an increased reliance on the distracting context information for response preparation under performance-contingent reward prospect both when this information was beneficial in most trials (Experiment 1) and when it was beneficial in the preceding trial only without predictive value for the upcoming trial (Experiment 2). This is in accordance with results from previous studies with modified versions of the AX-CPT that demonstrated increase usage of context cues under performance-contingent reward prospect even if these cues were no longer predictive or increased usage was maladaptive (Hefer & Dreisbach, 2016, 2017, 2020a). The present results generalize these findings by demonstrating a similar effect in a different paradigm and with a within-participants reward prospect manipulation. Thus, the results from our study support the conclusion that “[performance-contingent] reward prospect encourages the selective usage of any information that might be relevant for preparatory behavior […, even if] cue usage had never been experienced as adaptive in this context” (Hefer & Dreisbach, 2020a, p. 15). An important theoretical question remains as to whether this increased use of context information is a strategic adaptation due to expectations or a more automatic form of adaptation.

In this respect, the lack of a reversed Simon effect in mostly incongruent blocks seems important. In Experiment 1, where the context information was predictive of the upcoming congruency in a block, participants could have used this information to increase the focus on the distractor information and to prepare for a spatially non-corresponding response, resulting in a reversed Simon effect. Reversed Simon effects have been demonstrated before when participants experienced or merely expected mostly incongruent trials (e.g., Desender, 2018; Dreisbach et al., 2019; Logan & Zbrodoff, 1979; Stürmer et al., 2002). For example, Stürmer et al. (2002) showed, in line with dual route models of stimulus-response compatibility (Kornblum et al., 1990), two different mechanisms that contribute to the PCE in a lateralized readiness potentials (LRP) analysis of a Simon task. One is the suppression of early automatic response activation by distractor information in the direct route after an incongruent trial, which can explain reductions but not reversals of the Simon effect. This has been shown to underlie the CSE as well (see also, Stürmer & Leuthold, 2003). The other is an adaptation of the indirect route evidenced by earlier LRP onsets in frequent trial types, which suggests that participants make use of the distracting target position information to select the response (see also Logan & Zbrodoff, 1979, for a similar argument for divided attention between target and distractor information as an explanation for the PCE).

What might have prevented the latter mechanism in the present study are the use of rather short blocks of mostly (in)congruent trials in Experiment 1 and the lack of explicit instructions about the proportion congruency manipulation. Furthermore, proportion congruency always shifted within a given block toward an unbiased proportion congruency in the last 16 trials, thereby creating a global context of high volatility. While PCE-like control adaptations to proportion congruency have been demonstrated with even shorter block lengths (for a demonstration in a Stroop task, see Cohen-Shikora et al., 2018), a reversal of the Simon effect seems to require a less volatile context over a prolonged block of trials (Dreisbach et al., 2019; Logan & Zbrodoff, 1979; Stürmer et al., 2011) or explicit instructions on proportion congruency (Desender, 2018; Logan & Zbrodoff, 1979; see also Bugg et al., 2015, for an instruction-based PCE in a Stroop task on the first trial of a block). Control adaptations in contexts of high volatility have been shown to rely more on transient control adaptations (cf., Aben et al., 2017; Dey & Bugg, 2021), that is, the PCE under these conditions is due mostly to the influence of the immediately preceding trial and less on more distant trials. Thus, the PCE can be based on strategic adaptations to explicit expectations or more automatic adaptations based on stimulus-driven experience (Bugg & Crump, 2012). In the present paradigm, the PCE most likely resulted from the latter in terms of a cumulative effect of previous congruency and not from a strategic adaptation to explicit expectations. This could also explain the high similarity of results across Experiments 1 and 2.

Interestingly, our results indicated an asymmetry of the adaptation to congruency and the adaptation to incongruency. Performance-contingent reward prospect boosted the adaptation to congruent distractor information, whereas the adaptation of the congruency effect in mostly incongruent blocks (Experiment 1) or after incongruent trials (Experiment 2) did not differ between reward conditions. Instead, the Simon effect was equally reduced to a non-significant difference in all three conditions, a phenomenon that has been explained before with an increase in control to suppress the automatic response priming by the task-irrelevant target location (Stürmer et al., 2002). This seems to fit with observations by Jiménez and Méndez (2013, 2014), who investigated progressive CSEs in a Stroop task, that is, congruency effects depending on the sequence of more than one preceding trial. They found a cumulative increase of the congruency effect with an increasing number of preceding congruent trials. In contrast, the reduction of the congruency effect after incongruent trials seemed to have reached a maximum already after a single incongruent trial with no further reduction after consecutive incongruent trials. This suggests a differential functional role of conflicts and lack of conflict between target and distractor information in control adaptations in conflict tasks (cf., Berger et al., 2019; Lamers & Roelofs, 2011; Schlaghecken & Martini, 2012). To be more precise, while conflicts seem to instantly trigger an increased focus on the target and/or inhibition of the distractor, lack of conflict is followed by a more gradual relaxation of control. In the present study, only the latter process could be boosted by performance-contingent reward prospect, supposedly by increasing the usage of the congruent distractor information beyond a mere relaxation of control, while there was arguably no room for motivational modulation in the control adaptation following conflict.

### Performance-contingent versus non-contingent reward prospect

In the AX-CPT, oppositional effects have been found for non-contingent reward prospect compared to performance-contingent reward prospect (Fröber & Dreisbach, 2014, 2016a). While performance-contingent reward prospect increased the use of context information, non-contingent reward reduced the reliance on context information. This effect was comparable to the effect of positive affect induced via positive pictures, suggesting that non-contingent reward is primarily a positive affect manipulation, whereas performance-contingent reward is a motivational manipulation. In conflict studies with reward as feedback only, reward has also been shown to increase the CSE if rewards were performance-contingent (Braem et al., 2012; Stürmer et al., 2011), but to reduce the CSE if rewards were non-contingent (van Steenbergen et al., 2009, 2012; but see Stürmer et al., 2011, for a failed replication using a Simon task). This suggests that reward either reinforced or counteracted the aversive signal of a conflict depending on contingency (cf., Dreisbach & Fischer, 2015).

In the present study, performance under non-contingent reward prospect clearly differed from performance-contingent reward prospect, but had a comparable effect to the neutral control condition without reward prospect. Compared to motivational effects elicited by performance-contingent reward, non-contingent reward effects and other positive affect effects are rather subtle and can be easily abolished, for example, by motivational manipulations (Fröber & Dreisbach, 2014) or even by mere time on task (Hefer & Dreisbach, 2020b). Therefore, studies using a within-participants design in particular have failed to replicate typical positive affect effects before (e.g., Chiew & Braver, 2014). Since no study so far has employed both performance-contingent and non-contingent reward in a within-participants design, this procedure might be a boundary condition that prevents typical non-contingent reward effects. To speculate, the introduction of a social competition in the reward phase and the implementation of a performance-contingent reward manipulation in some of the blocks could have overshadowed the subtle effect of non-contingent reward.

It is remarkable that performance under non-contingent reward prospect was so similar to the neutral control condition. Participants were given rewards independently from performance, even for errors, and thus could have completely disengaged from the task by, for example, only pressing one response button as fast as possible. Instead, participants obviously continued to follow instructions. What might have encouraged them to stay on task is the feedback after each trial, which not only informed about reward receipt, but also about accuracy. A previous study (Fröber & Dreisbach, 2016a) already tested this hypothesis and continued to find high task engagement even if no accuracy feedback was given and non-contingent reward prospect was manipulated in a between-participants design, thereby having a prolonged impact on performance.

Taken together, the present results confirm that non-contingent reward prospect does not motivate participants to sloppy behavior. The expected induction of a relaxation of control in terms of less attenuation of distractor processing after (mostly) incongruent trials might, however, require a between-subjects design without a performance-contingent manipulation in the same experiment (Fröber & Dreisbach, 2014, 2016a; van Steenbergen et al., 2009, 2012). Maybe, the type of conflict task is furthermore important, since the influence of non-contingent reward feedback on the CSE was demonstrated in the Flanker task (van Steenbergen et al., 2009, 2012; Yamaguchi & Nishimura, 2019), but did not replicate with a Simon task before (Stürmer et al., 2011).

### Implications from drift diffusion modeling

In the present study, we used the DDM to enable a more fine-grained measure of cognitive processes beyond the behavioral variables mean RT and error rate and to directly investigate and control for the potential effect of the type of reward on speed-accuracy settings. We found that the PCE (Experiment 1) and the CSE (Experiment 2) were captured across experiments by the drift rate parameter. In mostly congruent blocks or following congruent trials, the drift rate was higher for congruent trials than incongruent trials, whereas the drift rate did not vary by congruency in mostly incongruent blocks or following incongruent trials (cf., Aschenbrenner, 2016). This fits with the idea that the drift rate in conflict tasks can be seen as accumulated evidence from a weighted sum of the task-irrelevant distractor information and the target information (cf., Logan & Zbrodoff, 1979; Ulrich et al., 2015).

In congruent trials, evidence from automatic activation by the distractor is in the same direction as the evidence by the target, whereas evidence accumulates in oppositional directions in case of an incongruent trial. Thus, in sum, the drift rate is smaller in incongruent than congruent trials. In mostly incongruent blocks (Experiment 1) or following incongruent trials (Experiment 2), the drift rate in the present study did not vary by congruency, which suggests an adjustment of the weights. As already argued before, the lack of a reversal of the Simon effect indicates a more automatic form of adaptation.^14^ The (repeated) experience of an incongruent trial seems to reduce the influence of the distractor, so that the difference between congruent and incongruent trials is attenuated. Such an attenuation of distractor information after incongruent trials fits with evidence from LRP analyses in the Simon task that demonstrate suppression of early distractor information (Stürmer et al., 2002; Stürmer & Leuthold, 2003). Due to the symmetrical data pattern of both increased drift rate in congruent trials and reduced drift rate in incongruent trials in mostly congruent blocks or following congruent trials, a possible explanation would be a boost of both facilitation and interference by the distractor by performance-contingent reward. Note, however, that a differentiation between facilitation and interference processes is not possible with the standard DDM. A recent study used a more complex version of the DDM, the “diffusion model for conflict tasks” (DMC; Ulrich et al., 2015), in an extended version to enable a dissociation between facilitation and interference effects, and found only a minor role of facilitation processes (Evans & Servant, 2022).

Furthermore, the DDM analysis revealed two distinct effects of performance-contingent reward prospect: (1) on the drift rate parameter and (2) on the boundary separation parameter. In the drift rate parameter, results mirrored the data pattern seen in the error rates. Performance-contingent reward prospect seemed to increase the weight given to the distractor information in mostly congruent blocks or following congruent trials. In addition, performance-contingent reward prospect lowered the threshold separation, suggesting a strategy shift with a less conservative response criterion. Notably, the reward manipulation did not affect other parameters of the diffusion model.

One might wonder whether it is just the speed focus induced by the performance-contingent reward condition that is responsible for the pattern of results rather than the performance-contingent reward per se. Notably, in many diffusion model studies that manipulated the focus on speed or accuracy (but not type of reward), not only threshold separation was affected, but also non-decision time and sometimes drift rate (e.g., Dutilh et al., 2019; Lerche & Voss, 2019; Rae et al., 2014; Voss et al., 2004). Not only threshold separation, but also non-decision time or drift rate are often lower in the speed compared to the accuracy condition. For example, Desender (2018) found a general reduction of the drift rate parameter with a speed instruction compared to an accuracy instruction in a Simon task. Using the DMC (Ulrich et al., 2015), Mittelstädt et al. (2021) likewise found that a speed instruction not only lowered the boundary separation, but also reduced the non-decision time and the drift rate of the target-based process (more so in a Simon task as compared to a Flanker task). In contrast, in our study, non-decision time was not modulated by the type of reward and the drift rate was comparable in all three reward conditions (Experiment 1) or even increased in the speeded performance-contingent reward condition (Experiment 2). Thus, while participants did use a more liberal response criterion under performance-contingent reward prospect, they showed signs of additional optimizations of performance in terms of the drift rate. Consistent with that interpretation, participants were generally faster but did not show generally increased error rates, but higher error rates specifically for incongruent trials in mostly congruent blocks (Experiment 1) or following congruent trials (Experiment 2). Thus, the reward manipulation was most likely not just a manipulation of the focus on speed or accuracy. Rather, we assume that the motivational component of the performance-contingent reward is responsible for both the main effect on threshold separation and the three-way interaction on drift rate. That affective and motivational effects can be captured in diffusion model parameters was already shown in some previous studies (e.g., Lerche et al., 2018; Lerche et al., 2019; Schuch & Pütz, 2021; Voss et al., 2013b). For example, in a study by Lerche et al. (2018), threshold separation was lower in a condition in which participants were given bogus, predominately positive performance feedback compared to a negative feedback condition.

While the standard DDM has been successfully applied before to model control adaptions like the CSE and the (instructed) PCE in different conflict tasks (Aschenbrenner, 2016; Desender, 2018; Schuch & Pütz, 2021), one of the more complex versions of DDMs that were designed specifically for conflict tasks (e.g., Hübner et al., 2010; Ulrich et al., 2015; White et al., 2011) would have been useful to get a more differentiated insight into changes of target- and distractor-based processes by performance-contingent reward prospect. Especially the DMC has been developed to analyze conflict tasks including the Simon task. Even more informative would have been the extended version of the DMC recently used by Evans and Servant (2022). Therein, not only target and distractor processing are captured in different model parameters, but also facilitation and interference effects within task-irrelevant distractor processing can be distinguished.

Critically, a parameter recovery study based on the DMC showed that the conflict-related parameters cannot be estimated reliably with small- to medium-sized trial numbers (White et al., 2018). The results of Evans and Servant (2022) are more promising, indicating that 200 trials per condition can be sufficient. However, in our experiments, the trial numbers per condition were clearly smaller (Experiment 1: 13–48 trials, Experiment 2: 25–36 trials). Thus, either only the main diffusion model parameters could be examined (which are also part of the standard DDM) or an experiment with many more trials would have been needed (which was not feasible in our repeated-measures design with three different reward conditions).

### Limitations and future directions

As already discussed, the PCE in our study was manipulated in a way that most likely resulted in relatively automatic and rather transient control adaptations (cf., Aben et al., 2017). Further experiments with extended blocks of the same proportion congruency and explicit instructions would be interesting to see if (non-)contingent reward prospect modulates more intentional and sustained control adaptations in the same way. Such a procedure would require a change to a between-participants design to prevent an unreasonable length. This might furthermore be advantageous for the investigation of non-contingent reward effects, which have been proven difficult to find with within-participants designs. Moreover, the number of trials per reward condition should be increased, which could enable the application of more complex versions of the DDM like the DMC, which provides a more differentiated analysis of target- and distractor-based processes (cf., Mittelstädt et al., 2021) and extended versions of the DMC that allow to distinguish between facilitation and interference effects (Evans & Servant, 2022) or are specialized to model control adaptation phenomena like the CSE (Luo & Proctor, 2022).

Another caveat of our study is that we investigated control adaptations in a conflict paradigm that was not optimized to differentiate between different underlying mechanisms of these adaptations. That is, control adaptations can rely on both higher-order cognitive control processes and low-level learning processes, while previous diffusion modeling of the CSE suggests that both kinds of processes are involved in the adaptation (Luo & Proctor, 2022). While we interpreted the present results from a cognitive control point of view, an alternative explanation could be that performance-contingent reward merely boosts associative learning processes like feature integration, contingency learning, or temporal learning (cf., Braem et al., 2019). To directly test which of these processes is affected by our reward prospect manipulation future studies should use a confound-minimized conflict paradigm (e.g., Weissman et al., 2014). Furthermore, some studies suggest qualitative differences between different conflict tasks (e.g., Aschenbrenner, 2016), while others suggest a common construct underlying various conflict tasks (e.g., Evans & Servant, 2022), so that future studies could also target the generalizability of the present finding to other conflict paradigms.

In general, more systematic research is needed on (non-)contingent reward effects. The literature on reward effects is characterized by heterogeneous results, which in part is due to a huge variety in procedures (contingent vs. non-contingent, reward prospect vs. feedback, pure reward motivation vs. mixed motivation by reward and loss), often accompanied by little differentiation in nomenclature. Future studies should clearly state which aspect of reward (cf., Notebaert & Braem, 2016) is targeted by a study, and how this is implemented. Furthermore, more fine-grained analyses with the DDM or more complex models seem to be a valuable addition in reward research to clarify which cognitive processes are affected by reward, and whether reward effects are qualitatively different from mere speed-accuracy effects or not.

## Conclusion

In two well-powered experiments, we investigated how performance (non-)contingent reward modulates context processing in a conflict task. We found that specifically performance-contingent reward prospect increased the congruency effect in the error rates especially in mostly congruent blocks (Experiment 1) or after congruent trials (Experiment 2). This effect was captured by the drift rate parameter of the diffusion model, suggesting that the use of congruent distractor information was boosted by performance-contingent reward, thereby reducing the error rate in congruent trials but also increasing the error rate in incongruent trials. In contrast, control adaptations to (repeated) incongruent trials were not further modulated by the type of reward. The Simon effect in the drift rate was reduced to zero, but not reversed, suggesting that responses were based on target information alone and the impact of the distractor information was attenuated.

## Footnotes

1 We are aware of the ongoing controversies about the proportion congruency effect and the congruency sequence effect as control adaptation phenomena. There are various alternative explanations for these phenomena that are not all based on the assumption of cognitive control processes. However, the PCE and the CSE can still be found in so-called confound-free variants that minimize or exclude the contribution of alternative explanations based on low-level features alone. In this literature review, we use the term *control adaptations* as a generic term irrespective of the specific underlying mechanism. We will come back to various theoretical explanations for control adaptation phenomena in the *General discussion*.

2 Using different approaches, the issue of speed-accuracy trade-offs in reward studies has been addressed before with (1) post hoc analyses, for example, by Chiew and Braver (2013) and Fröber and Dreisbach (2016a), with (2) procedures that implemented response thresholds in all conditions, for example, by Shen and Chun (2011) and Fröber and Dreisbach (2016b), or with (3) procedures that disentangled reward prospect effects from the actual, speeded task execution, for example, by Fröber et al. (2019) and Fröber and Dreisbach (2021). In sum, cumulative evidence suggests that reward prospect impacts performance beyond a mere speed-accuracy trade-off.

3 Note that sporadic negative RTs are encountered in several studies that used *lab.js*, most likely due to premature responses (for a discussion of this issue, see the lab.js project Slack channel).

4 Note that the substantial range in trial numbers per condition is attributable to the unequal proportions of congruent and incongruent trials in mostly congruent or mostly incongruent blocks.

5 Additionally, we examined whether the method of data collection (lab vs. online) moderated the Proportion Congruency × Congruency N interaction effect. Neither for mean RT nor for error rate was there a significant three-way interaction (both *p*s > .309).

6 Like for the baseline phase, the Proportion Congruency × Congruency N interaction effect was not moderated by the data collection method (lab vs. online; all *p*s > .146). The Reward × Proportion Congruency × Congruency N interaction was not moderated for the drift rate (*p* = .193), non-decision time (*p* = .228), and error rate (*p* = .490). For mean RT, there was a small, significant four-way interaction effect (*p* = .037), which, however, should not be overinterpreted given that it was not expected and that it was only present in one of several dependent variables.

7 In additional analyses, for mean RT, error rate, and drift rate, we examined the Simon effect (i.e., the effect of Congruency N) separately for the two conditions of Proportion Congruency. In the mostly congruent condition, the Simon effect was significant for all three reward conditions (all *p*s <.001). In the mostly incongruent condition, on the other hand, there was no significant difference in either of the reward conditions (all *p*s > .475).

8 The effect of Type of Reward on threshold separation was not moderated by the data collection method (lab vs. online; *p* = .742).

9 For the behavioral variables mean RT and error rate we additionally conducted analyses with inclusion of these two participants, and the inclusion did not change the pattern of results.

10 Additionally, we examined whether the method of data collection (lab vs. online) moderated the Congruency N-1 × Congruency N interaction effect. For mean RT (*p* < .001), there was a significant three-way interaction. Most importantly, if the lab and online sample are examined in separate ANOVAs, the two-way interaction effect remained significant.

11 In terms of mean RT, the Congruency N-1 × Congruency N interaction effect was moderated by the data collection method (lab vs. online, *p* < .01). Separate ANOVAs revealed a significant Congruency N-1 × Congruency N interaction in both samples. Regarding all the other dependent variables, there was no significant three-way interaction effect (all *p*s > .243). Furthermore, there was no significant data collection method × Reward × Congruency N-1 × Congruency N interaction (all *p*s > .161).

12 In additional analyses, for mean RT, error rate, and drift rate, we examined the Simon effect (i.e., the effect of Congruency N) separately for the two conditions of Congruency N-1. For trials with preceding congruent trials, the Simon effect was significant for all three reward conditions (all *p*s <.001). For trials with preceding incongruent trials, on the other hand, there was no significant difference in all reward conditions (all *p*s > .281).

13 The effect of Type of Reward on threshold separation was not moderated by the data collection method (lab vs. online; *p* = .903).

14 Desender (2018) demonstrated a reversed Simon effect in the drift rate, when an explicit expectation for mostly incongruent trials is induced. This suggests that the automatic activation by the distracting location information can be intentionally overridden, resulting in a higher drift rate in incongruent trials and a lower drift rate in congruent trials.

## Declarations

### Open practices statement

The raw data for both experiments are available at https://osf.io/f29td/.
